# Bird nests as botanical time capsules: DNA barcoding identifies the contents of contemporary and historical nests

**DOI:** 10.1371/journal.pone.0257624

**Published:** 2021-10-06

**Authors:** Alex Rinkert, Tracy M. Misiewicz, Benjamin E. Carter, Aleezah Salmaan, Justen B. Whittall

**Affiliations:** 1 Department of Biological Sciences, San José State University, San José, CA, United States of America; 2 Department of Microbiology and Plant Biology, University of Oklahoma, Norman, OK, United States of America; 3 Department of Biology, Santa Clara University, Santa Clara, CA, United States of America; University of Hyogo, JAPAN

## Abstract

Bird nests in natural history collections are an abundant yet vastly underutilized source of genetic information. We sequenced the nuclear ribosomal internal transcribed spacer to identify plant species used as nest material in two contemporary (2003 and 2018) and two historical (both 1915) nest specimens constructed by Song Sparrows (*Melospiza melodia*) and Savannah Sparrows (*Passerculus sandwichensis*). A total of 13 (22%) samples yielded single, strong bands that could be identified using GenBank resources: six plants (Angiospermae), six green algae (Chlorophyta), and one ciliate (Ciliophora). Two native plant species identified in the nests included *Festuca microstachys*, which was introduced to the nest collection site by restoration practitioners, and *Rosa californica*, identified in a nest collected from a lost habitat that existed about 100 years ago. Successful sequencing was correlated with higher sample mass and DNA quality, suggesting future studies should select larger pieces of contiguous material from nests and materials that appear to have been fresh when incorporated into the nest. This molecular approach was used to distinguish plant species that were not visually identifiable, and did not require disassembling the nest specimens as is a traditional practice with nest material studies. The many thousands of nest specimens in natural history collections hold great promise as sources of genetic information to address myriad ecological questions.

## Introduction

Bird nest specimens are abundant in many natural history collections, yet they are vastly underutilized as a tool for answering questions in ecology [1]. Worldwide there are nearly 60,000 bird nest specimens archived in natural history collections that span the past 250 years [2, 3]. As new technologies emerge, museum specimens are increasingly being used as a source of genetic information [4]. The spatial, temporal, and taxonomic representation of nest specimens makes them an attractive and untapped source of ecological data [1], and nest material can be used to study a variety of ecological topics such as architectural camouflage, chemical defense from pathogens and parasites, intraspecific signaling, seed dispersal, and mammal biogeography [5–11]. The increasing ubiquity of artificial materials, especially plastics, in the nests of some species has also demonstrated the utility of learning about pollution and land use change in the environment through materials incorporated into bird nests (see [12]). Despite many birds using plants as nest material, few studies attempt to make inferences from nests made primarily of plant material, possibly because of the challenges associated with identifying the plant species from this material type.

Identifying plant material in bird nests is hindered because pieces of plant material often do not contain taxonomically useful and distinctive structures. The number of plant species and amount of materials that can be identified varies tremendously (see [6, 13–15]), and the distinctiveness of the plant material incorporated into a nest undoubtedly determines how much material in the nest can be identified. For example, many passerines, such as New World sparrows (Emberizidae), use dried grasses as nest material [16]. This plant material typically does not contain the reproductive structures that species identifications hinge upon, rendering the principal material in these nests visually unidentifiable. The age of nests that can be studied is a further limitation, as while studies describing the types of materials in freshly collected nests are typically disassembled and examined *ex situ* (e.g., [8, 12, 15]), this destructive sampling practice is avoided in natural history collections (e.g., [5, 17]) likely because of the historical value of the specimens.

A number of advances in molecular biology have facilitated the genetic analysis of historical specimens. Next generation sequencing technologies are compatible with fragmented and degraded DNA that is typical of museum specimens [18, 19] and the development of primers that target short amplicons have led to successful species identifications from degraded plant material, including ancient material dating back 10,000 years [20, 21]. The techniques used to obtain DNA from preserved plant specimens have overcome various challenges with specimen preparation and taxon-specific traits (see [22]), as well as restrictions for destructively sampling historical specimens [23]. Some of these challenges are no different than those that would be encountered with bird nest specimens, albeit the quality of the materials may be poorer as nests are often made of dead plant materials, unlike plant specimens which are typically collected from a living organism.

We investigated the utility of using the nuclear ribosomal internal transcribed spacer (ITS) barcode [24] to identify plant species from bird nest material. We identified several plant species from bird nests and identified sample characteristics that can be used as predictors for sequencing success. Finally, we provide recommendations for using a molecular approach to identify plant species from nest material.

## Methods

### Nest sampling

**Fig 1:**
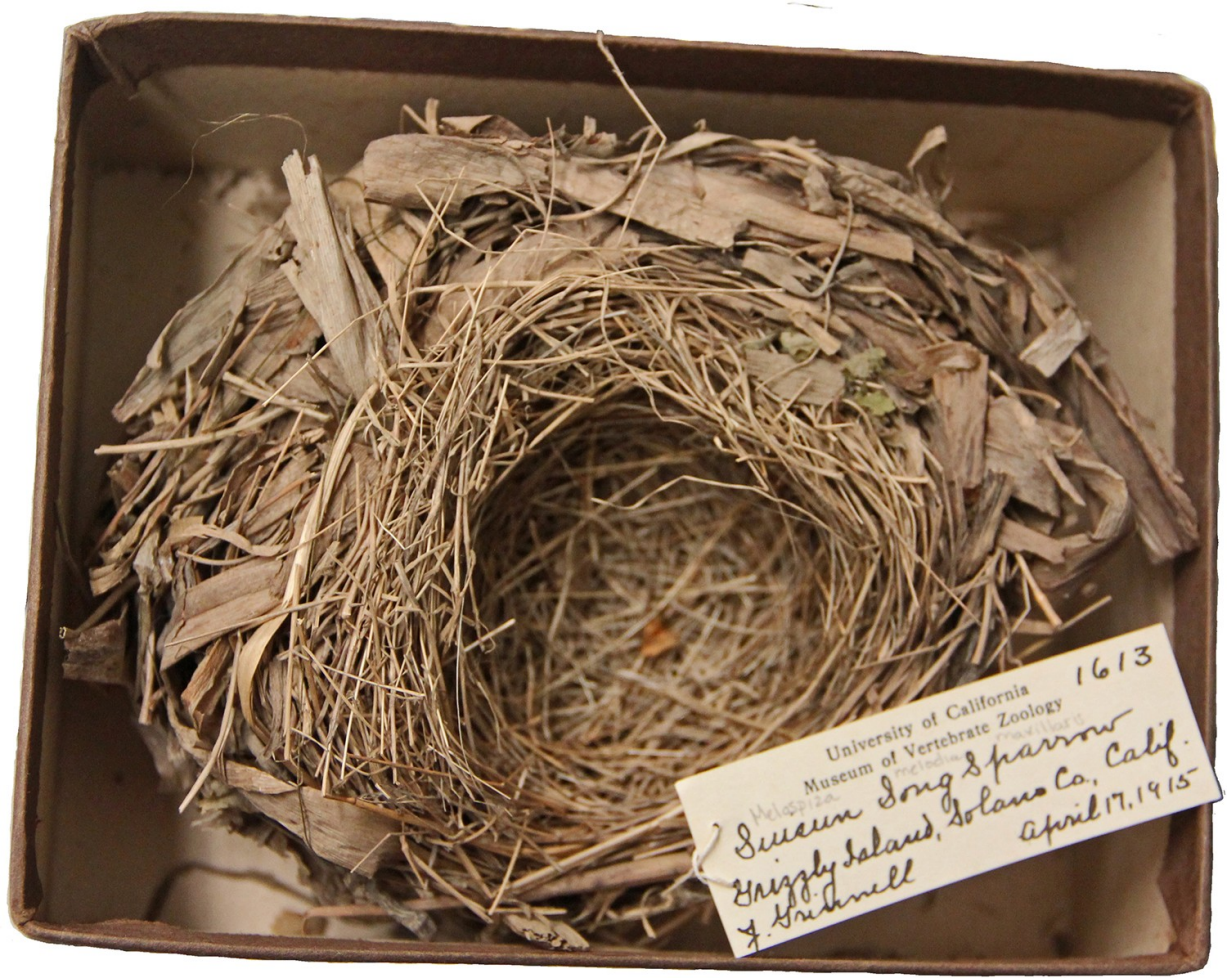
Plant material was sampled from the structure (coarse outer material) and lining (fine inner material) of this Song Sparrow (*Melospiza melodia maxillaris*) nest specimen (MVZ1:Egg:1613).

**Fig 2:**
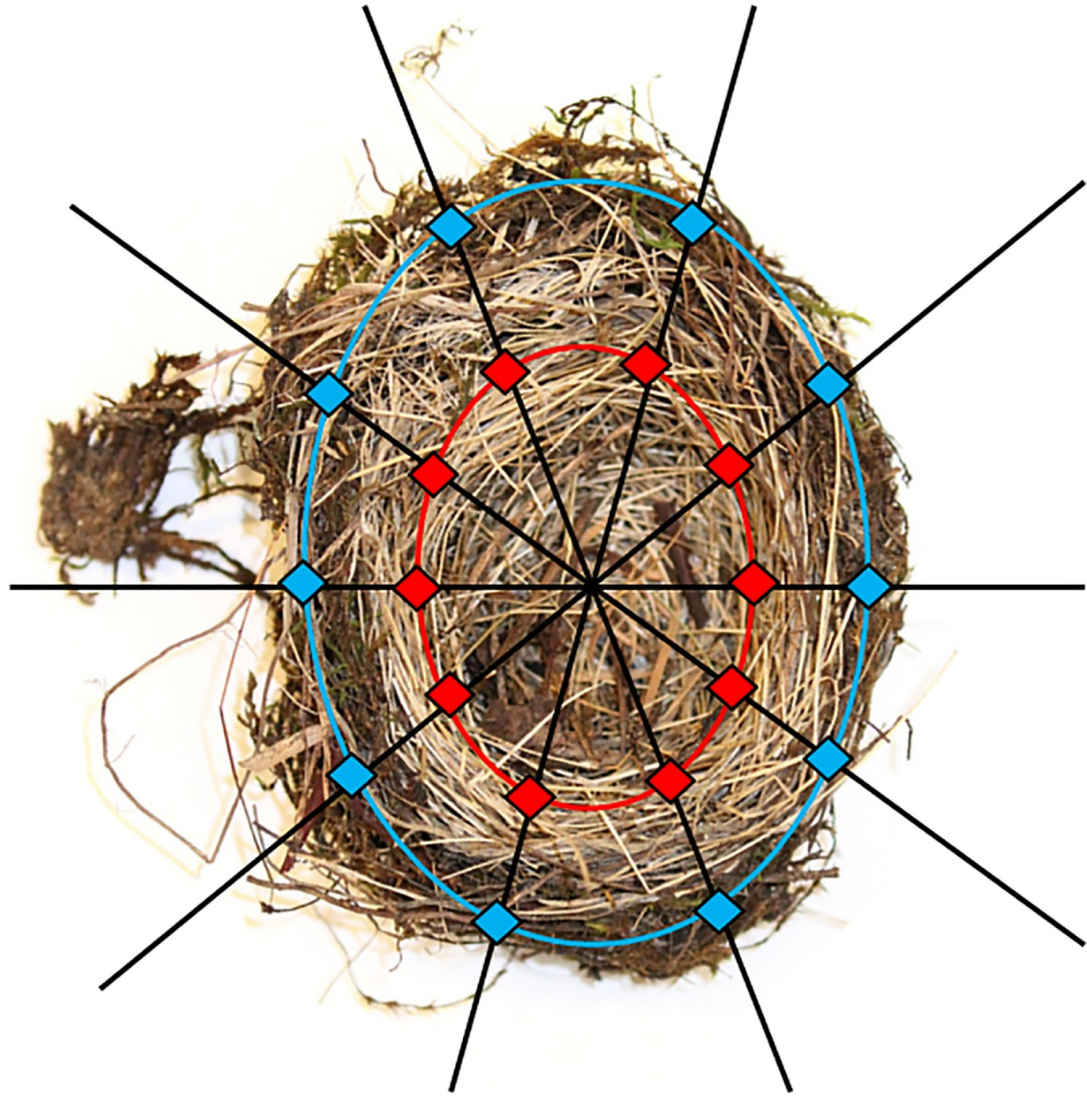
Samples were systematically selected from the structure (blue oval) and lining (red oval) at equally spaced locations (diamonds).

Four nests were chosen to represent two collection eras: historical and contemporary. Two nests collected in 1915 were considered “historical” nests, while two nests, one collected in 2003 and another in 2018, were considered “contemporary.” Three nests selected for sampling were built by Song Sparrows (*Melospiza melodia*; one contemporary and two historical), while one nest was built by a Savannah Sparrow (*Passerculus sandwichensis*; one contemporary) (Table 1, Fig 1). These two sparrow species build an open-cup nest with an outer structure variably made of coarse dried grass, fibers and woody material, and an inner lining made of finer dried grass, hair, feathers, and other soft material [25–27]. Samples were systematically selected from the structure and lining of the two historical nests (MVZ:Egg:1611, MVZ:Egg:1613; Table 1, Fig 2) by selecting materials at equally spaced intervals from the two concentric layers, the structure and lining, of the nest. Initial sampling in the contemporary nest specimens SFBBO1 and SFBBO2 was less systematic and intensive as these specimens were used to test the approach before sampling historical nests, however the materials selected were representative of the materials in the structure and lining of each specimen. Each sample constituted a single contiguous piece of plant material from the nest. All samples were photographed and weighed before DNA was extracted (S1 and S2 Figs, S1 Table). Care was taken to prevent cross-contamination between samples by using instruments initially sterilized with 70% EtOH, cleaning instruments between sampling with sterile delicate task tissue, using different instruments for each nest, and ensuring no loose fragments of materials were inadvertently included in a sample.

**Table 1:** Bird nest specimens selected for sampling.

Specimen	Collection Year	Collection Location	Taxon	Samples		
				*Structure*	*Lining*	*Total*
MVZ1:Egg:1611^1^	1915	Bay Farm Island, Alameda County, CA	*Melospiza melodia pusillula*	9	9	18
MVZ1:Egg:1613^1^	1915	Grizzly Island, Solano County, CA	*Melospiza melodia maxillaris*	9	5	14
SFBBO1^2^	2003	Coyote Creek, Santa Clara County, CA	*Melospiza melodia* ^3^	2	2	4
SFBBO2^2^	2018	Bair Island, San Mateo County, CA	*Passerculus sandwichensis bryanti*	13	11	24
			**Total**	**33**	**27**	**60**

^1^ Museum of Vertebrate Zoology, University of California, Berkeley, USA.

^2^ San Francisco Bay Bird Observatory private collection.

^3^ Nest collected in the contact zone of *M*. *m*. *gouldii* and *M*. *m*. *pusillula* where they are known to intergrade (Demers et al. 2012). The subspecies of SFBBO1 could not be determined.

### DNA extraction and amplification

DNA was extracted following a modified protocol of the NucleoSpin Plant II kit (Macherey & Nagel, Bethlehem, PA). Samples were homogenized using a Biospec BeadBeater. The NucleoSpin Plant II protocol (using buffer PL1) was followed for extracting genomic DNA from plants, with each extraction being eluted once with 50 uL of Buffer PE. DNA concentration was quantified with a Qubit fluorometer using the Qubit dsDNA BR Assay Kit (Thermo Fisher Scientific, Waltham, MA), and purity was estimated from the 260nm/280nm ratio measured on a NanoDrop 1000 spectrophotometer (Thermo Fisher Scientific, Waltham, MA). For each DNA extraction and PCR, a separate, negative control with no nest material was included to aid in detecting contamination. Throughout the molecular procedures, special precautions were taken to avoid contamination including using sterile tubes, filtered pipette tips, freshly prepared reagents, and pipettes not previously exposed to plant DNA. Samples from historical nests were physically isolated from contemporary nest samples in storage and lab procedures to prevent cross-sample contamination [28].

PCR reactions were performed in 50 μL volume containing 2 μL of DNA template, 25mM MgCl2, 1x Standard Taq Buffer, 2.5mM of each dNTP, 1uM of the forward and reverse primers [24], and 1 unit of Taq DNA polymerase (New England Biolabs, Ipswich, MA). All samples were tested for amplification of the entire ITS region (ITS-p5f/ITS-u4r), yet this rarely worked so ITS1 and ITS2 were targeted separately with primer combinations ITS-p5f/ITS-u2r (ITS1) and ITS-p3f/ITS-u4r (ITS2). Thermal cycling conditions began with a 2 min denaturation at 92°C and then 34 cycles of the following: 1 min denaturation at 94°C, 45 sec annealing at 55°C, and 45 sec extension at 72°C. Lastly, there was a final extension for 5 min at 72°C. Following amplification, 5 μL of each PCR product was run on a 1% agarose gel and all samples producing a single band were submitted for sequencing. A single band visible on the gel was considered sequencing success for that sample regardless of species identification in downstream analyses. These samples were purified using shrimp alkaline phosphatase and then sequenced in both directions using ITS primers on an ABI 3730 (Applied Biosystems) at Sequetech Corp. (Mountain View, CA).

### Bioinformatics

Raw sequences were manually trimmed to remove the primer and any low-quality bases at the ends using Geneious v7 [29]. When available, forward and reverse sequences were assembled into a single contiguous sequence applying IUPAC ambiguity codes wherever any superimposed nucleotide additivity patterns were detected [30]. The sequence length and HQ% in Geneious were recorded after processing the sequence.

Sequences were initially analyzed using BLAST [31]; the Megablast [32] search within BLASTn was used to query the non-redundant database in GenBank. The following were extracted from the top BLAST match(es) based on the Max Score metric: expect (E) value, % coverage, and % sequence identity. Samples that BLAST identified as a plant were further analyzed using a genetic distances approach and a phylogenetic analysis. To produce alignments, the top 100 BLAST hits were downloaded to Geneious and automatically aligned with the sample using the Geneious aligner. Alignments were trimmed to the length of the sample and the most similar sequence was identified by the percent genetic identity. All phylogenetic reconstructions were conducted using the maximum likelihood framework implemented in RAxML [33] and used the GTR+CAT+I algorithm with 1000 bootstrap replicates set up as a plugin in Geneious v7 [29].

Correlations between sample and sequence characteristics with sequencing success and taxonomic group (i.e., plants, green algae, and ciliates) were tested using a series of non-parametric significance tests. A Wilcoxon rank sum test was used to test for correlations between sample dry weight, DNA concentration, or DNA purity (260nm/280nm) with sequencing success. For samples with sequencing success, a Wilcoxon rank sum test was also used to test for correlations between sequence quality (HQ%) and taxa (i.e., plant, green algae). All statistical analyses were carried out using R [34], and *p* < 0.05 was considered statistically significant.

## Results

Thirteen (22%) of the 60 total samples produced a single strong PCR product of the expected size range for either ITS1 (n = 1), ITS2 (n = 11), or the entire ITS region (n = 1) (Table 2). Eleven (39%) of the 28 samples from the contemporary nests (SFBBO1, SFBBO2) produced a usable sequence compared to only two (6%) of the 32 samples from the two historical nests (MVZ:EGG:1611, MVZ:EGG:1613).

**Table 2:** Taxonomic identifications of samples.

Sample	BLAST^1^	Genetic Distance	Maximum Likelihood Phylogenetic Analysis	BLAST			Genetic Distance Value (%)	Maximum Likelihood Bootstrap Value (%)
				*E-value*	*Query Coverage*	*Identity*		
8–3	*Festuca eskia/ovina*	*Festuca microstachys* ^2^	*Festuca microstachys*	1.00E-171	100	97.00	99.396	99
8–6	*Cardamine hirsuta*	*Cardamine hirsuta*	*Cardamine hirsuta*	0	100	100.00	100	100
8–8^3^	Triticeae	*Elymus triticoides/cinereus/ambiguus*	*Elymus triticoides/cinereus/ambiguus*	6.00E-140	100	100.00	29–0	65
8–13^4^	*Aristerostoma* sp.	-	-	6.00E-150	100	97.48	-	-
9–16	*Elymus triticoides/cinereus/ambiguus*	*Elymus triticoides/cinereus/ambiguus*	*Elymus triticoides/cinereus/ambiguus*	3.00E-167	100	100.00	100	65
9–20	Chlorophyta	-	-	1.00E-22	21	93.83	-	-
10–16	*Pseudostichococcus monallantoides*	-	-	4.00E-77	99	84.08	-	-
12–5	*Gayralia oxysperma*	-	-	7.00E-114	99	92.26	-	-
12–10	*Pseudendoclonium arthopyreniae*	-	-	2.00E-64	100	82.94	-	-
12–12	*Tupiella speciosa*	-	-	9.00E-45	80	95.08	-	-
12–17	*Salicornia pacifica/neei*	*Salicornia pacifica/perennis*	*Salicornia pacifica/perennis*	0	88	99.72	100	75
17–8^5^	*Dilabifilum arthopyreniae*	-	-	9.00E-154	99	92.35	-	-
29–4^6^	NA	*Rosa carolina*	*Rosa californica*	0	100	99.45	99.631	64

^1^ BLAST identification based on the maximum score. Multiple names indicate tied maximum score.

^2^ *Festuca pampeana* was the second-closest to *F*. *microstachys* in genetic distance at 96.995%.

^3^ 8–8 and 9–16 are 100% identical in the area they overlap. 9–16 provenance possibly uncertain?.

^4^ The ITS1 region is reported for 8–13; the ITS2 region is reported for all other samples.

^5^ 17–8 has two sequences: p3f HQ% 60.9, bp 345; u4r HQ% 63.0, bp 338.

^6^ Values and identifications for 29–4 are based on the ITS1 and ITS2 region.

Of the thirteen successfully sequenced samples, six (46%) were identified as flowering plants (Angiospermae), six (46%) were identified as green algae (Chlorophyta), and one (8%) was identified as a ciliate (Ciliophora) (Table 2). The two historical nest identifications included one plant and one green alga, while identifications from the contemporary nests consisted of the remaining five plant samples, five green algae samples, and one ciliate. The six samples identified as angiosperms represented four families: Amaranthaceae, Brassicaceae, Poaceae, and Rosaceae. In all but one of the plant samples, the bioinformatic analyses consistently identified the following taxa to genus or species: *Cardamine hirsuta* (S3 Fig), *Festuca* (S4 Fig), *Leymus* (S5 Fig), *Salicornia* (S6 Fig), and *Rosa californica* (S7 Fig). The three genera where species identification varied between the three bioinformatic analyses pertained to two or three species (Table 2). When these species were filtered by their likelihood of being present in the San Francisco Bay estuary based on their known range, five distinct species of plants remained.The plant identifications to genus and species were based on three metrics: BLAST E-value, genetic identity, and phylogenetic analysis bootstrap support. The average E-value for the top BLAST hit of the six plant sequences was 5.09x10^-27^ (range 0–6.0x10^-140^) with an average of 98% query coverage and 99.36% average sequence identity (Table 2). The two alignment-based methods, genetic distances and phylogenetic analysis, produced similar taxonomic identifications. The top percent identity from the distance matrix based on the alignment of the top 100 BLAST hits for each of the six plant sequences ranged from 99.4% to 100% (average = 99.8%; Table 2). The maximum likelihood phylogenetic analyses paired each plant sample with one or more GenBank sequences with an average bootstrap support of 78% (range 65%–100%).

**Fig 3:**
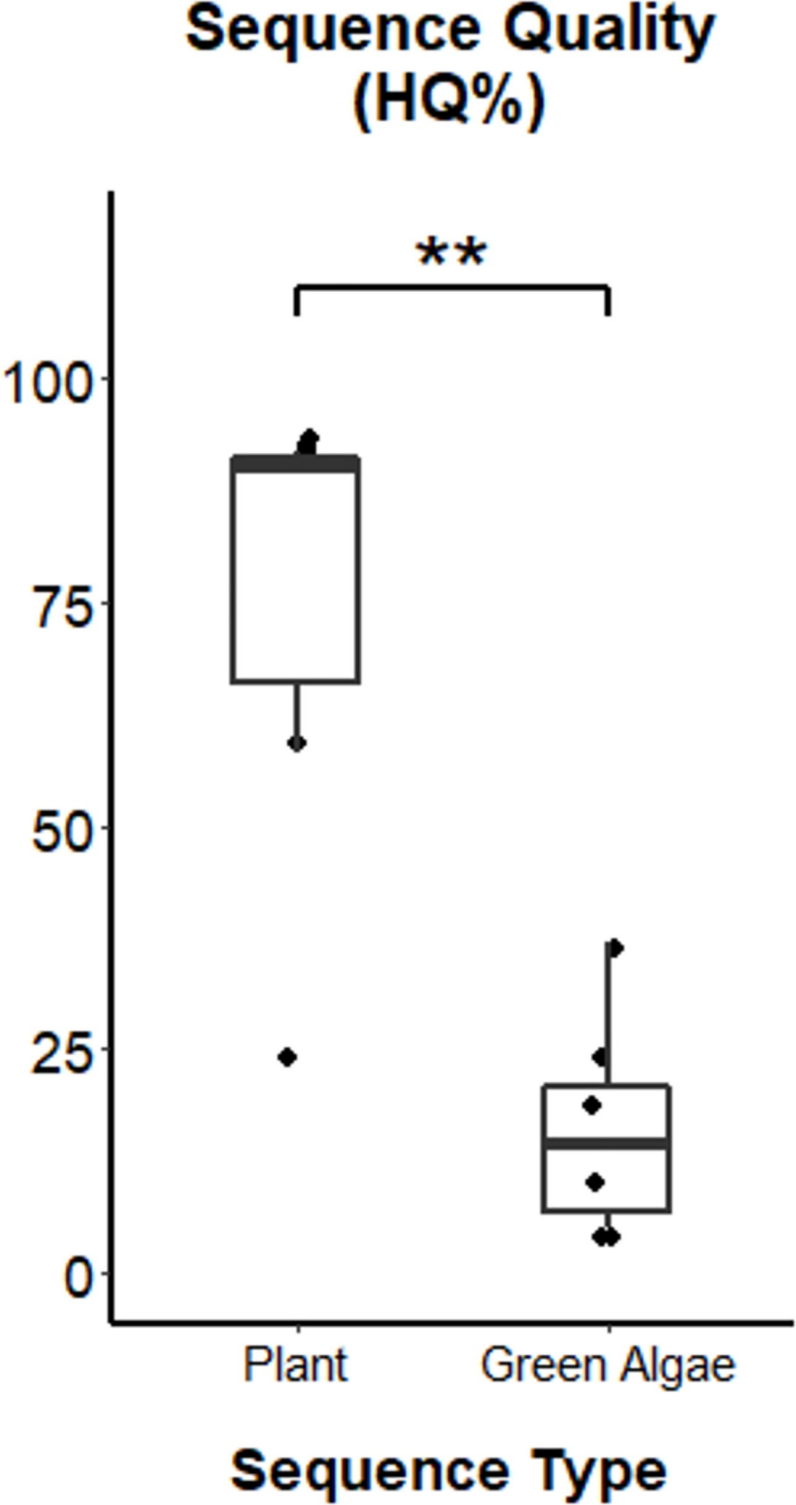
Difference in sequence quality (HQ%) between samples identified as plant and green algae. Significant correlations are indicated as follows: ** p < 0.01.

The seven samples identified as non-angiosperms were more difficult to confidently identify because the samples had relatively low sequence quality (Table 3). Sequence quality was significantly higher for plant sequences (median HQ% = 90.8) than green algae sequences (median HQ% = 16.9%) (Wilcoxon rank sum test, W = 1, p = 0.011; Table 3, Fig 3). At least three of the six samples of green algae were unique and represented different clades. The average E-value for the top BLAST hit was 2.79x10^-10^ (range 1.0x10^-22^–9.0x10^-154^) with an average of 83% query coverage and 90.09% average sequence identity (Table 2). The two alignment-based methods, genetic distances and phylogenetic analysis, also indicated low confidence in these identifications. The one ciliate sample was more confidently identified by BLAST than the green algae samples based on the lower E-value (6.00x10^-140^), and higher query coverage (97.47%) and sequence identity (97.48%).

**Table 3:** Characteristics of successfully sequenced samples.

Sample	Taxonomic Group	Dry Mass (mg)	DNA Concentration (ng/uL)	DNA Purity (260/280)	HQ%	bp
8–3	Plant	11.2	9.12	1.73	92.0	364
8–6	Plant	32.2	20.6	1.71	90.0	351
8–8	Plant	16.1	4.2	1.69	25.9	108
8–13	Ciliate	62.2	32.7	1.75	24.3	317
9–16	Plant	1.0^1^	2.73	-12.35	58.0	324
9–20	Green algae	1.0^1^	0.972	1.42	5.0	357
10–16	Green algae	10.0^1^	13.9	1.45	11.8	313
12–5	Green algae	40.0^1^	76.8	1.86	16.9	301
12–10	Green algae	40.0^1^	127	1.84	22.0	286
12–12	Green algae	20.0^1^	0	1.09	36.9	149
12–17	Plant	40.0^1^	62	1.78	91.7	407
17–8	Green algae	14.2	11.7	1.59		
29–4	Plant	17.4	0.884	1.31	89.9	719

^1^ Some initial experiments called for predetermined amounts of dry mass.

**Fig 4:**
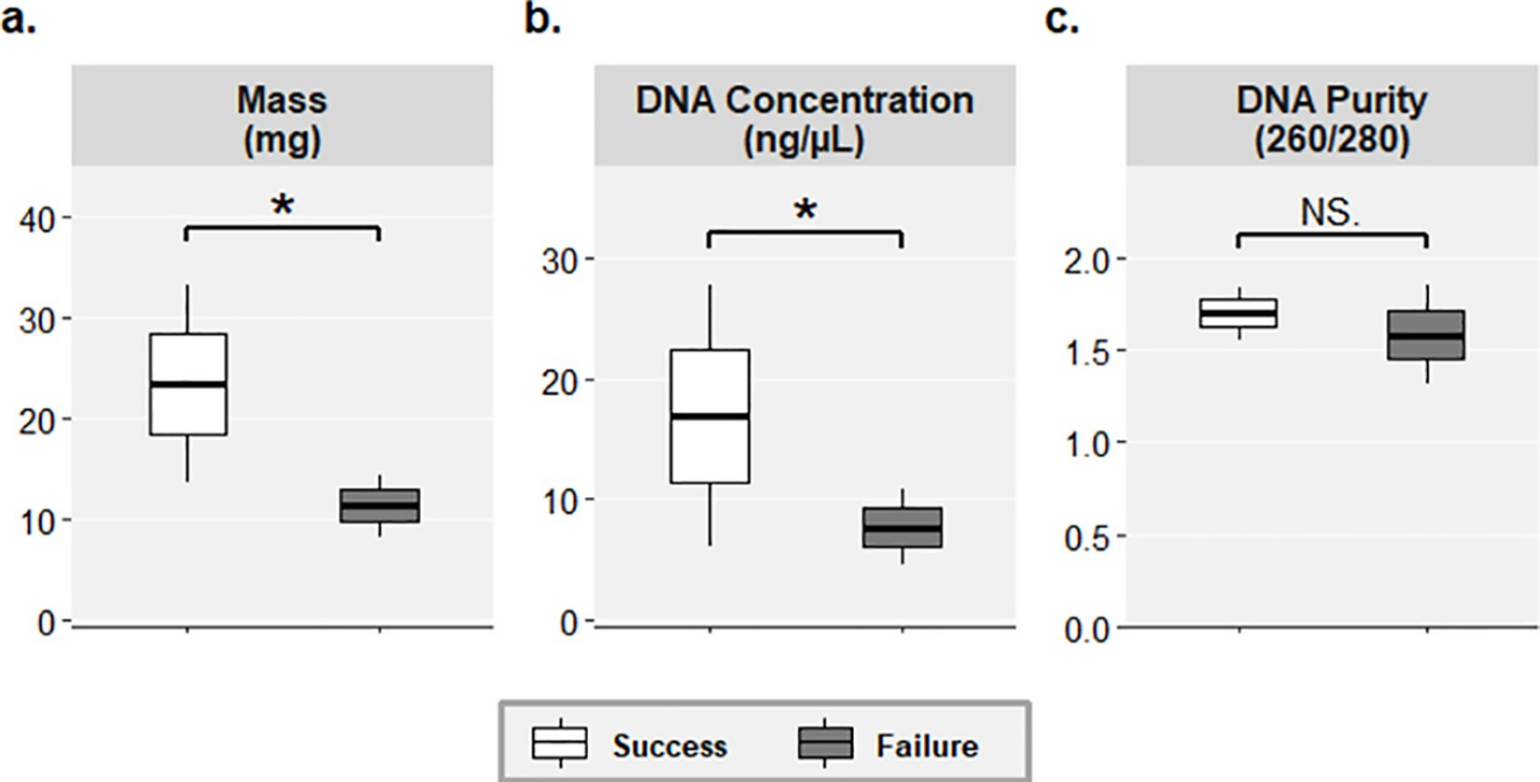
Differences in sample characteristics (**a.** dry mass, **b.** DNA concentration, and **c.** DNA purity) between samples with sequencing success or failure. Significant correlations are indicated as follows: * p < 0.05, ** p < 0.01, NS. = not statistically significant.

Three sample characteristics (dry mass, DNA concentration, and DNA purity) were tested for correlations with sequencing success. The samples successfully sequenced had higher dry mass than samples that did not amplify (Wilcoxon rank sum test, W = 182.5, p = 0.006; Fig 4A). Likewise, the DNA concentration in samples successfully sequenced was significantly higher than in those that did not amplify (Wilcoxon rank sum test, W = 160, p = 0.011; Fig 4B). There was no significant difference in the DNA purity (260/280 ratio) between samples that were successfully sequenced and those that failed to amplify (Wilcoxon rank sum test, W = 182.5, p = 0.554; Fig 4C).

## Discussion

### Genetic information from bird nests

Thirteen samples representing historical and contemporary collection eras were successfully sequenced from bird nest specimens, and sequences could be identified using existing references in GenBank. While most samples lacked recognizable structures that could be used to visually identify the plant species, by using a molecular approach we identified six plants that likely represent five species. Specimens in natural history collections often serve as a source of genetic information to answer ecological and evolutionary questions (e.g,. [18, 19]), however we are unaware of plant material from bird nest specimens previously being used for this purpose.

### Revealing the historical ecology of lost habitats

Nest specimens are botanical time capsules containing a physical representation of the environment where they were collected. Plants identified in historical nests can unlock new information about the ecology of historical environments that have since changed. We identified a native rose (*Rosa*) in an historical nest (MVZ1:Egg:1613) collected in a habitat that has since been lost to dramatic landscape changes. While some Song Sparrows are known to use *Rosa* shrubs as a nest substrate [35], the *R*. *californica* sample selected from the nest specimen was a fine stem woven between the lining and structure so it is unlikely to be part of the nest substrate but rather intentionally incorporated into the nest. Furthermore, no other apparent *Rosa* material was noted in the nest.

The historical nest specimen was constructed by a tidal marsh-dwelling Song Sparrow, *M*. *m*. *maxillaris*, which nests in marshes adjacent to transitional habitat in the San Francisco Bay estuary [36]. The nests of this taxon are lined with grasses and contain other plant material that does not grow in tidally influenced marshes where they nest, but rather originates from the adjacent transitional habitat (A. Rinkert pers. obs.). Transitional habitat, the ecological gradient between the tidal marshes and uplands [37], was lost by the early 1900s as a result of major landscape changes [38, 39], and consequently, very little is known about the former native plant community. The only historical description of the plant community in transitional habitat [38] mentioned ten plant species, including *R*. *californica*, growing in relict patches between 1913–1915. The *R*. *california* identified in the historical nest specimen confirms this plant species was historically part of transitional habitat. Further sampling and sequencing from historical nest specimens of Song Sparrows from the San Francisco Bay estuary could reconstruct transitional habitat more completely by revealing additional plant species once occurring there.

While the genetic identity and phylogenetic analyses support the identification as *R*. *californica* (S7 Fig), the genus *Rosa* is incompletely represented in GenBank. Only two species of native California *Rosa*—*R*. *californica* and *R*. *woodsii*—are represented by ITS sequences in GenBank (GenBank Release 241: December 15 2020). Furthermore, *R*. *californica* is apparently weakly differentiated at the trnL chloroplast loci from *R*. *nutkana* [40], another native California *Rosa* that is known to occur near marshes in the estuary and does not have an ITS reference sequence in GenBank [41, 42]. Incomplete representation of species in GenBank may prove to be an initial limitation to identifying species from nest materials, however the existing GenBank resources were able to identify all six plant species to genus, and species identifications are possible with the development of reference sequences in target genera (S1 Protocol).

### Bird nests as a tool for restoration

Nest material can be used to help determine the success of habitat restoration. Restoration success of a native plant community is rarely evaluated, especially on a holistic scale [43]. We identified *Festuca microstachys*, a California native [44] incorporated into a nest specimen collected in a habitat restoration area at Bair Island, CA (Table 2, S5 Fig). This restoration area was formerly a decommissioned salt pond with narrow levee slopes covered in exotic vegetation. Prior to restoration at this site in 2016, there were no occurrences of *F*. *microstachys* at Bair Island in plant observation databases [42, 45] and the only native species observed on these levee slopes prior to restoration were *Baccharis pilularis* and *Salicornia pacifica* (A. Rinkert pers. obs.). In 2017 restoration practitioners introduced a broad suite of native species, including *F*. *microstachys*, to this site to establish a native plant community [46, 47; S2 Table]. The nest specimen SFBBO2 was subsequently collected 2 m from the restoration area in 2018. Considering the apparent absence of *F*. *microstachys* prior to restoration, we conclude this nest material originated from a plant introduced by restoration practitioners, which demonstrates birds immediately utilize native plant species for nest building. We propose comparing the nest materials of birds before and after restoration to evaluate the recovery of ecological interactions, as well as determine plant species preferences for nest material. Restoration areas typically offer a broad suite of native plants which, combined with vegetation surveys at the site, can offer insight into the selectiveness of certain plants for nest materials, further refining restoration efforts in the future to better benefit species that select specific materials for their nest.

### Circumventing challenges with studying nest material

A major advantage of using a molecular approach to identify plants in bird nests is that the materials do not need to retain any structures that could be used to visually identify the species. Two nondescript pieces of grass culms were identified to the tribe Triticeae, with the likely species being *Elymus triticoides* (S5 Fig, Table 2), which is native to the collection site [44]. The bioinformatic analyses could not distinguish the two sample sequences from two other species (*E*. *ambiguus* and *E*. *cinereus*) within *Elymus*, but they are either unrecorded in California or occur very rarely in the San Francisco Bay estuary [42, 48], so we conclude these two samples were *E*. *triticoides*, a common species at the collection site [44, A. Rinkert pers. obs.]. The taxonomic resolution of this species identification would not be possible to achieve with visual examination as the materials do not retain structures that are used to distinguish these three species in *Elymus* [48]. A broader range of nest types could be sampled by using a molecular approach as species identification would not hinge on the material retaining identifiable structures, and the nest specimen would not need to be disassembled in contrast with traditional practices.

### Considerations for sanger sequencing of nest material

#### Utilizing historical and contemporary nest specimens

Historical nest specimens in natural history collections are an invaluable source of genetic information, however the preservation conditions of these historical specimens warrant consideration when sampling. The preservation technique and storage of many historical nest specimens may be unknown. Cross-contamination between specimens stored in the same physical space may have occurred, and for this reason we recommend only selecting materials that are incorporated into the architecture of the nest, and not loose on the exterior of the nest or detached materials accompanying the specimen. Historical nest specimens were also typically subjected or inadvertently exposed to various types of chemical treatments (see [49]). These chemicals could interfere with the molecular processes used to obtain DNA from historical nest specimens (e.g., [50, 51]). When possible, we recommend choosing specimens to sample from that are known to not have been treated with chemicals as a precautionary measure.

The same considerations for historical nest specimens generally apply to contemporary nest. Studies calling for the collection of nest specimens should store nests separately in sterile containers and the nests should not be chemically treated. Instead, nests should be frozen or dried, both of which preserve DNA in woody plant material [52]. Lastly, identifying the species or type of nest substrate can help with interpreting species identifications from the nest. If materials from the nest substrate are inadvertently sampled and identified, these results should be interpreted cautiously as the materials may not have been deliberately added to the nest.

#### Identifying appropriate specimens and materials for sampling

Nests that are made with degraded plant material introduce challenges with obtaining high quality sequences as low yields and environmentally caused damage and contamination can interfere with molecular procedures, sharing similar challenges with ancient DNA [53, 54]. We recommend larger pieces of contiguous material be selected from nest specimens to maximize the yield of DNA obtained; if no larger pieces of material exist, then multiple samples of similar morphology can be pooled to obtain an equivalent critical mass. Higher dry sample mass and DNA concentration were predictors of sequencing success, likely because of the proportional increase in DNA yield with mass.

Selecting specific pieces of material based on their morphological or visual characteristics may also lead to higher sequencing success. Only one green leaf was observed in the four nests we sampled, and the sample of this material yielded the *Rosa californica* identification. While green leaves have little effect on the DNA obtained from historical herbarium specimens [55], the leaf from the nest specimen may have been freshly collected and more quickly desiccated, and therefore allowing the green color and DNA to be better preserved. Leaves are ubiquitously used as a source of DNA for botanical studies [52] making this material in nests most advantageous to sample, however new techniques can help overcome some of the physical and chemical challenges with obtaining DNA from woody stems and aromatic material, that are sometimes used by birds as nest material. Selecting specific portions of woody stems and ensuring complete desiccation can maximize the yield of DNA in woody stems [52], while modifying standard CTAB extraction protocols to better handle undesirable chemical compounds can improve DNA quality [56].

Sanger sequencing may be most effective on nest specimens made of certain types of materials. Nests constructed of green, fresh material may produce samples with higher yields of DNA and resultantly higher sequencing success, as this material is most similar to what is conventionally used in botanical studies [52]. Species such as the European Starling (*Sturnus vulgaris*), weaverbirds (Ploceidae), and some swifts (Cypseloidinae) collect fresh nest material from living plants, and in some cases build a nest that is a living organism itself [9, 57–59], making these ideal nest types for studies utilizing Sanger sequencing.

#### Identifying a genetic marker

Considering over 90% of candidate plant species from the nest collection sites were represented in GenBank (S2 Table), we suggest this approach has widespread utility in other comparably sampled habitats. While GenBank serves as an adequate reference library, housing over 379,637 non-redundant angiosperm sequences (GenBank Release 241: accessed February 15 2021), confident species identification using ITS alone can be limited by several factors. The lack of sequence variation among closely related species, inconsistent phylogenetic signals at the tips of the tree (e.g., *Elymus*), and incomplete sampling of taxonomic groups all limit the taxonomic certainty and resolution of identification. For the latter, we demonstrate with *R*. *californica* that even if the taxonomic group is incompletely sampled, GenBank resources provide an approximate identification (e.g., to genus) that can be narrowed when supplemented with reference sequences from herbarium specimens or fresh field collections, such as *R*. *californica* (e.g., S1 Protocol). Including additional, rapidly evolving and widely sampled loci such as cpDNA introns/spacers or nuclear introns from conserved orthologous genes [60–62] may improve confidence in species identifications. The high percentage of plant species at the collection sites with ITS reference in GenBank helps assure species identifications are accurate, and should be considered as a prerequisite for selecting nest specimens to sample.

#### Distinguishing between target and spurious results

Spurious results may be produced when using a highly sensitive molecular procedure to amplify extremely low yields of DNA (see [63, 64]). While we targeted the kingdom Plantae, we amplified and identified six taxa of green algae and one ciliate (Table 2). The five green algae sequences we could identify to species using BLAST are multicellular organisms known to occur in marine and brackish water [65–68], while the one ciliate identified is also known to be a marine species [69]. While none of the green algae are specifically known to occur in the San Francisco Bay estuary, where all four nest specimens were collected, it is plausible they are naturalized as the marine ecosystem in this estuary is known to be highly invaded [70]. All green algae sequences had high BLAST E-values and all but two (samples 12–10 and 17–8) represent different lineages [65–68]. We postulate these green algae and the ciliate were incidentally deposited on the nest material as all nest specimens were collected from areas with some tidal influence or stream flow, instead of originating from lab contamination.

### Future directions

Molecularly identifying plants in nest specimens is a viable method of identifying species, however the type of nest material and overarching goals of the study will determine whether the Sanger sequencing approach is appropriate. Sampling across a wider variety of nest types will further ascertain what species build nests that are most conducive to obtaining DNA through this method, and whether climate and nest material type factor into the preservation of the materials.

There is a wealth of ecological and evolutionary questions that can be addressed with DNA obtained from nest specimens, especially with the temporal and spatial breadth of specimens available in natural history collections. Sampling historical nest specimens in conjunction with contemporary specimens can be used to determine how nest material preferences have changed following land use changes, and can even identify shifts in nest material preference as a result of climate change [71]. For some species, preferences for specific types of nest materials differ across their range [57, 72]. Further ascertaining geographical differences in nest material preferences may reveal patterns corresponding with the relative availability of specific types of nest materials, and perhaps identify whether the availability of these materials may limit their distribution. The nest material used by some species may not vary spatially or temporally, and identifying and understanding the evolution of these strong preferences may further help conservation efforts.

Beyond plants, other communities could even be reconstructed from DNA obtained from nest specimens. Invertebrate and mammal remains identified in contemporary bird nests have confirmed the presence of species in areas where they were previously not known to occur (e.g., [8, 73]). Finally, the application of third generation genome sequencing technologies applied to new materials like bird nests will greatly expand the prospects of identifying ecological connections in past and present ecosystems.

## Supporting information

S1 Fig Sample photos from two contemporary nest specimens. (TIF)

S2 Fig Sample photos from two historical nest specimens. (TIF)

S3 Fig Phylogenetic relationship of nest sample 8–6 with *Cardamine hirsuta*. (TIF)

S4 Fig Phylogenetic relationship of nest sample 8–3 with *Festuca microstachys*. (TIF)

S5 Fig Phylogenetic relationship of nest sample 8–8 and 9–16 with *Elymus* (*Leymus*) *triticoides*. (JPG)

S6 Fig Phylogenetic relationship of nest sample 12–17 with *Salicornia pacifica*. (JPG)

S7 Fig Phylogenetic relationship of nest sample 29–4 with *Rosa californica*. (TIF)

S1 Table Characteristics of nest specimen samples. Samples with successful PCR results are indicated in bold.
(DOCX)

S2 Table Candidate plant species from nest collection sites and availability of GenBank sequences. (DOCX)

S1 Protocol Creating an ITS reference sequence for *Rosa californica*. (DOCX)
