# Supplementary figures and images for: Bird nests as botanical time capsules: DNA barcoding identifies the contents of contemporary and historical nests
Source: PLoS One. 2021 Oct 6;16(10):e0257624. doi: 10.1371/journal.pone.0257624 (PMC8494352; doi:10.1371/journal.pone.0257624)

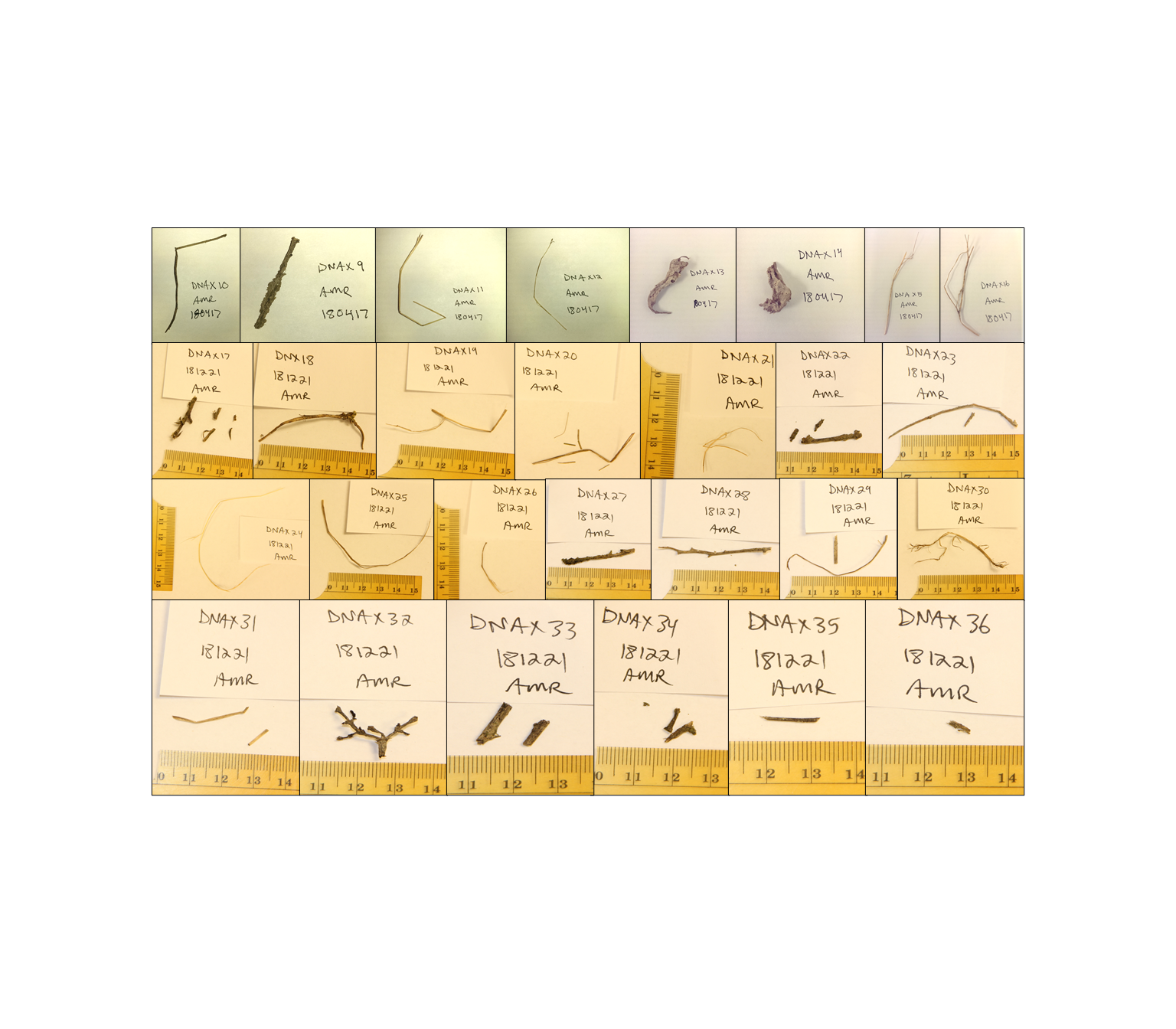

Supplement: S1 Fig — (TIF) [file pone.0257624.s001.tif]

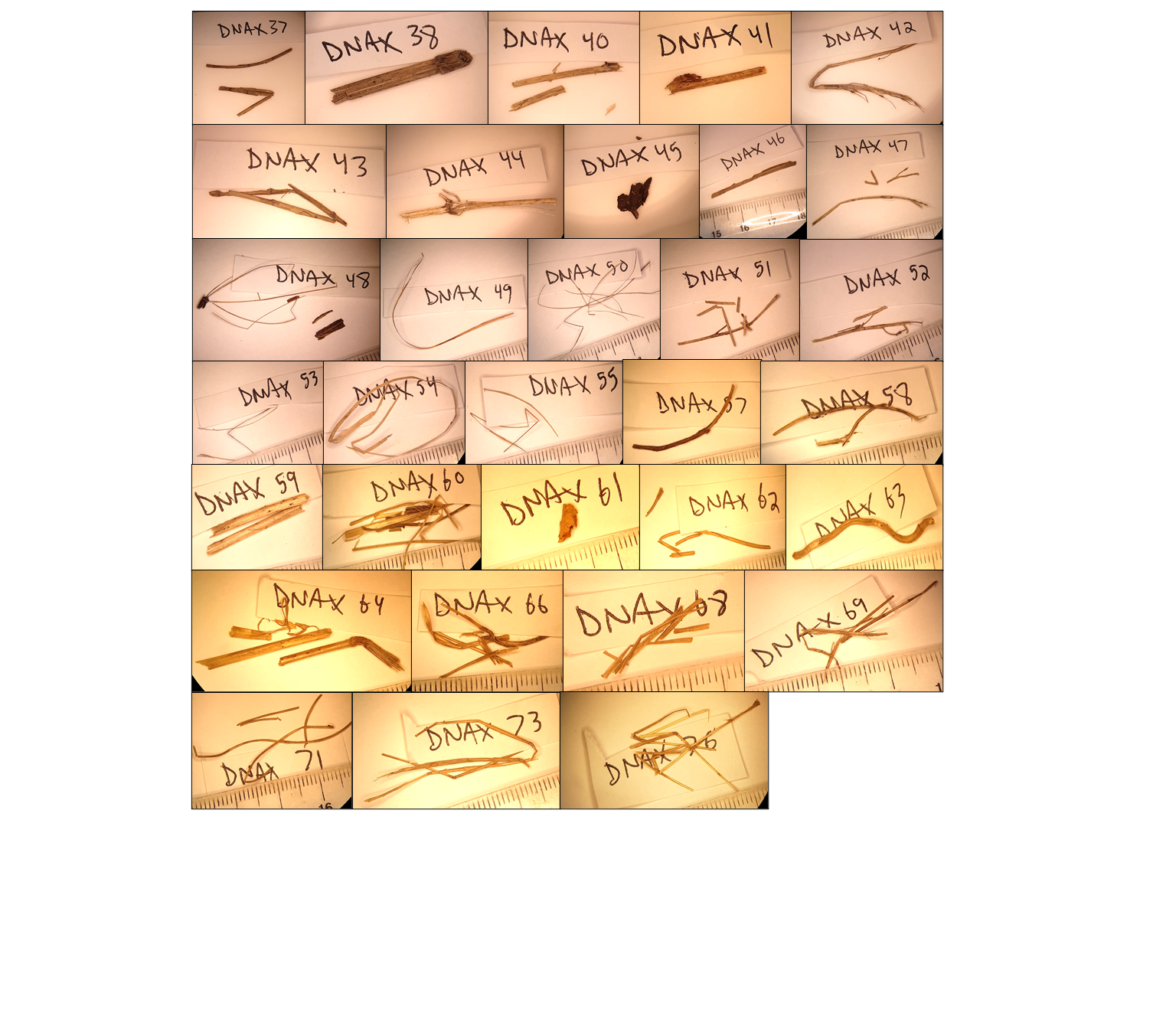

Supplement: S2 Fig — (TIF) [file pone.0257624.s002.tif]

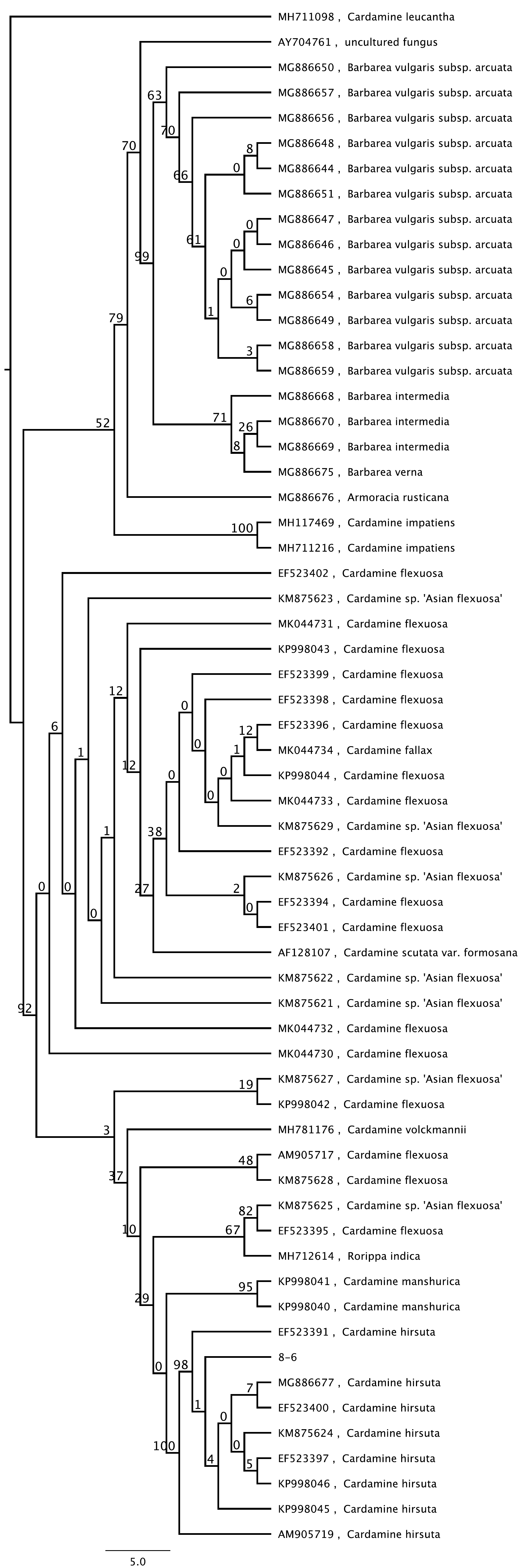

Supplement: S3 Fig — (TIF) [file pone.0257624.s003.tif]

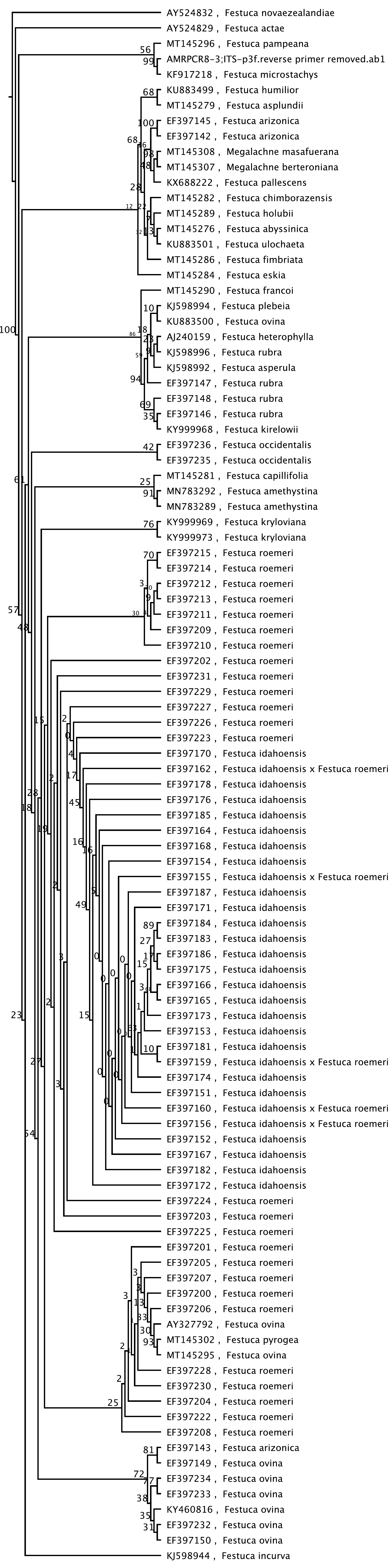

Supplement: S4 Fig — (TIF) [file pone.0257624.s004.tif]

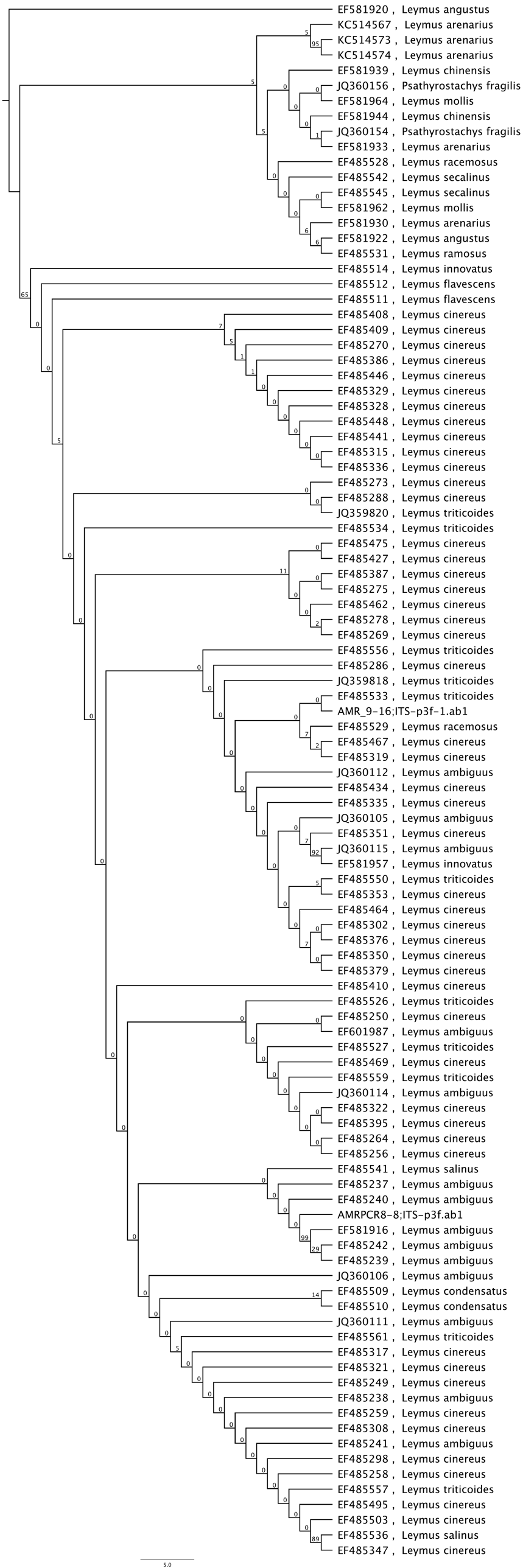

Supplement: S5 Fig — (JPG) [file pone.0257624.s005.jpg]

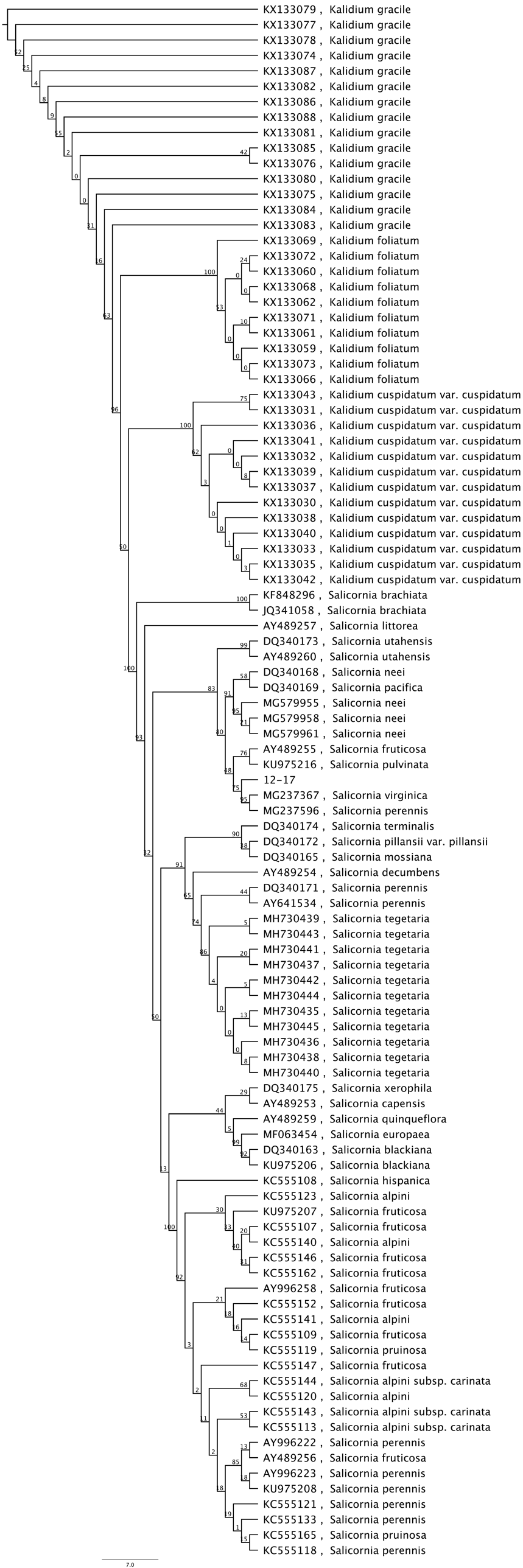

Supplement: S6 Fig — (JPG) [file pone.0257624.s006.jpg]

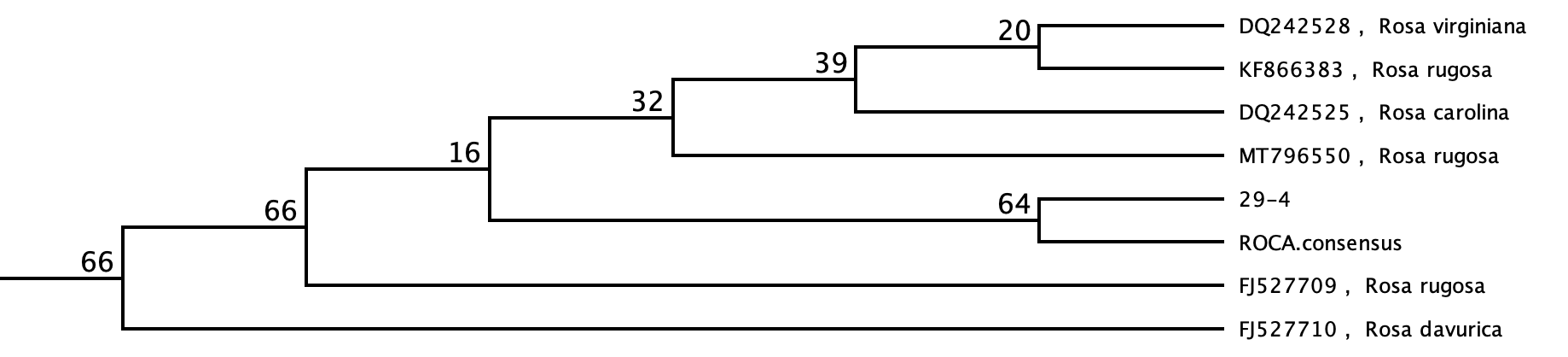

Supplement: S7 Fig — (TIF) [file pone.0257624.s007.tif]
