# Supplementary material for: Bird nests as botanical time capsules: DNA barcoding identifies the contents of contemporary and historical nests
Source: PLoS One. 2021 Oct 6;16(10):e0257624. doi: 10.1371/journal.pone.0257624 (PMC8494352; doi:10.1371/journal.pone.0257624)
Supplement: S1 Table — Samples with successful PCR results are indicated in bold. (DOCX) [file pone.0257624.s008.docx]

**S3 Table.** **Characteristics of nest specimen samples.** Samples with successful PCR results are indicated in **bold**.

| **DNAX** | **Nest** | **Species^1^** | **Nest Placement** | **Extraction Method** | **Starting Mass (mg)** | **DNA Concentration (ng/uL)** | **DNA Purity (260/280)** |
| --- | --- | --- | --- | --- | --- | --- | --- |
| 9 | SFBBO2 | SAVS | Structure | Column | 53.4 | 21.1 | 1.66 |
| 10 | SFBBO2 | SAVS | Structure | Column | 7.7 | 9.76 | 1.53 |
| **11** | **SFBBO2** | **SAVS** | **Lining** | **Column** | **11.2** | **9.12** | **1.73** |
| 12 | SFBBO2 | SAVS | Lining | Column | 12.7 | NA | 1.09 |
| **13** | **SFBBO1** | **SOSP** | **Structure** | **Column** | **62.2** | **32.7** | **1.75** |
| **14** | **SFBBO1** | **SOSP** | **Structure** | **Column** | **32.2** | **20.6** | **1.71** |
| 15 | SFBBO1 | SOSP | Lining | Column | 22.9 | 2.81 | 1.39 |
| **16** | **SFBBO1** | **SOSP** | **Lining** | **Column** | **16.1** | **4.2** | **1.69** |
| **17** | **SFBBO2** | **SAVS** | **Structure** | **Column** | **40.0** | **127** | **1.84** |
| 18 | SFBBO2 | SAVS | Structure | Column | 20.0 | 45.2 | 1.98 |
| 19 | SFBBO2 | SAVS | Lining | Column | 5.0 | 14.4 | 2.76 |
| 20 | SFBBO2 | SAVS | Lining | Column | 10.0 | 16.1 | 2.06 |
| 21 | SFBBO2 | SAVS | Lining | Column | 1.0 | 0.616 | -1.01 |
| 22 | SFBBO2 | SAVS | Structure | Column | 40.0 | 93.26 | 1.95 |
| **23** | **SFBBO2** | **SAVS** | **Structure** | **Column** | **20.0** | **27.1** | **2.28** |
| 24 | SFBBO2 | SAVS | Lining | Column | 10.0 | 2.5 | 5.18 |
| 25 | SFBBO2 | SAVS | Lining | Column | 5.0 | 19.2 | 2.18 |
| 26 | SFBBO2 | SAVS | Lining | Column | 1.0 | 0.552 | -1.2 |
| **27** | **SFBBO2** | **SAVS** | **Structure** | **Column** | **40.0** | **76.8** | **1.86** |
| 28 | SFBBO2 | SAVS | Structure | Column | 20.0 | 33.4 | 1.65 |
| 29 | SFBBO2 | SAVS | Lining | Column | 5.0 | 15.6 | 2.89 |
| **30** | **SFBBO2** | **SAVS** | **Lining** | **Column** | **10.0** | **13.9** | **1.45** |
| **31** | **SFBBO2** | **SAVS** | **Lining** | **Column** | **1.0** | **2.73** | **-12.35** |
| **32** | **SFBBO2** | **SAVS** | **Structure** | **Column** | **40.0** | **62** | **1.78** |
| 33 | SFBBO2 | SAVS | Structure | Column | 20.0 | 36 | 1.61 |
| 34 | SFBBO2 | SAVS | Structure | Column | 10.0 | 14.6 | 1.76 |
| 35 | SFBBO2 | SAVS | Structure | Column | 5.0 | 1.37 | 1.16 |
| **36** | **SFBBO2** | **SAVS** | **Structure** | **Column** | **1.0** | **0.972** | **1.42** |
| 37 | MVZ1:Egg:1611 | SOSP | Structure | Column | 17.7 | 26 | 1.76 |
| 38 | MVZ1:Egg:1611 | SOSP | Structure | Column | 30.2 | 4.72 | 1.82 |
| 40 | MVZ1:Egg:1611 | SOSP | Structure | SDS | 11.6 | 3.91 | 1.86 |
| 41 | MVZ1:Egg:1611 | SOSP | Structure | Column | 13.2 | 0 | 1.37 |
| 42 | MVZ1:Egg:1611 | SOSP | Structure | SDS | 9.8 | 15.4 | 1.58 |
| **43** | **MVZ1:Egg:1611** | **SOSP** | **Structure** | **Column** | **14.2** | **11.7** | **1.59** |
| 44 | MVZ1:Egg:1611 | SOSP | Structure | SDS | 12.3 | 1.46 | 1.57 |
| 45 | MVZ1:Egg:1611 | SOSP | Structure | Column | 5.1 | 0 | 1.82 |
| 46 | MVZ1:Egg:1611 | SOSP | Structure | SDS | 8.2 | 14.6 | 1.48 |
| 47 | MVZ1:Egg:1611 | SOSP | Lining | SDS | 4.4 | 9.96 | 1.46 |
| 48 | MVZ1:Egg:1611 | SOSP | Lining | SDS | 5.0 | 2.95 | 1.22 |
| 49 | MVZ1:Egg:1611 | SOSP | Lining | Column | 16.4 | 0 | 3.02 |
| 50 | MVZ1:Egg:1611 | SOSP | Lining | SDS | 2.0 | 0 | 1.35 |
| 51 | MVZ1:Egg:1611 | SOSP | Lining | Column | 6.0 | 2.42 | 1.56 |
| 52 | MVZ1:Egg:1611 | SOSP | Lining | Column | 8.6 | 0.416 | 1.53 |
| 53 | MVZ1:Egg:1611 | SOSP | Lining | SDS | 5.9 | 0 | 2.12 |
| 54 | MVZ1:Egg:1611 | SOSP | Lining | Column | 34.3 | 0 | 1.06 |
| 55 | MVZ1:Egg:1611 | SOSP | Lining | SDS | 1.8 | 0 | 1.06 |
| 57 | MVZ1:Egg:1613 | SOSP | Structure | Column | 7.0 | 1.28 | 1.46 |
| 58 | MVZ1:Egg:1613 | SOSP | Structure | SDS | 5.0 | 1.56 | 1.62 |
| 59 | MVZ1:Egg:1613 | SOSP | Structure | SDS | 11.3 | 1.81 | 2.09 |
| 60 | MVZ1:Egg:1613 | SOSP | Structure | Column | 4.2 | 18.7 | 1.58 |
| **61** | **MVZ1:Egg:1613** | **SOSP** | **Structure** | **Column** | **17.4** | **0.884** | **1.31** |
| 62 | MVZ1:Egg:1613 | SOSP | Structure | Column | 13.3 | 3.15 | 1.01 |
| 63 | MVZ1:Egg:1613 | SOSP | Structure | SDS | 8.6 | 0.584 | 0.58 |
| 64 | MVZ1:Egg:1613 | SOSP | Structure | Column | 16.6 | 4.52 | 1.46 |
| 66 | MVZ1:Egg:1613 | SOSP | Structure | Column | 6.6 | 0 | 1.21 |
| 68 | MVZ1:Egg:1613 | SOSP | Lining | SDS | 1.8 | 0.56 | 1.36 |
| 69 | MVZ1:Egg:1613 | SOSP | Lining | SDS | 4.5 | 0.836 | 1.76 |
| 71 | MVZ1:Egg:1613 | SOSP | Lining | SDS | 0.8 | 0.616 | -5.09 |
| 73 | MVZ1:Egg:1613 | SOSP | Lining | SDS | 8.0 | 1.65 | 1.81 |
| 76 | MVZ1:Egg:1613 | SOSP | Lining | Column | 6.6 | 0 | 0.73 |

^1^ SOSP = Song Sparrow (*M. melodia*); SAVS = Savannah Sparrow (*P. sandwichensis*).
