# Supplementary material for: Bird nests as botanical time capsules: DNA barcoding identifies the contents of contemporary and historical nests
Source: PLoS One. 2021 Oct 6;16(10):e0257624. doi: 10.1371/journal.pone.0257624 (PMC8494352; doi:10.1371/journal.pone.0257624)
Supplement: S2 Table — (DOCX) [file pone.0257624.s009.docx]

**S10 Table.** Candidate plant species from nest collection sites and availability of GenBank sequences.

| **Plant Species** | **Guild** | **Provenance** | **ITS^1^** | **Chloroplast^2^** |
| --- | --- | --- | --- | --- |
| *Achillea millefolium* | Forb | Native | Y | Y |
| *Acmispon americanus* | Forb | Native | N | Y |
| *Agrostis avenacea* | Grass | Exotic | N | Y |
| *Ambrosia psilostachya* | Forb | Native | Y | Y |
| *Amsinckia intermedia* | Forb | Native | Y | Y |
| *Amsinckia menziesii* | Forb | Native | Y | Y |
| *Anagallis arvensis* | Forb | Exotic | Y | Y |
| *Artemisia californica* | Forb | Native | Y | Y |
| *Artemisia douglasiana* | Forb | Native | Y | Y |
| *Arthrocnemum subterminale* | Forb | Native | Y | Y |
| *Atriplex prostrata* | Forb | Exotic | Y | Y |
| *Atriplex semibaccata* | Forb | Exotic | Y | Y |
| *Avena barbata* | Grass | Exotic | Y | Y |
| *Avena fatua* | Grass | Exotic | Y | Y |
| *Baccharis glutinosa* | Forb | Native | N | Y |
| *Baccharis pilularis* | Forb | Native | Y | Y |
| *Bassia hyssopifolia* | Forb | Exotic | Y | Y |
| *Beta vulgaris* | Forb | Exotic | Y | Y |
| *Brassica nigra* | Forb | Exotic | Y | Y |
| *Brassica rapa* | Forb | Exotic | Y | Y |
| *Bromus carinatus* | Grass | Native | Y | Y |
| *Bromus diandrus* | Grass | Exotic | Y | Y |
| *Bromus hordeaceus* | Grass | Exotic | Y | Y |
| *Bromus madritensis* | Grass | Exotic | Y | Y |
| *Calendula officinalis* | Forb | Exotic | Y | Y |
| *Capsella bursa-pastoris* | Forb | Exotic | Y | Y |
| *Carduus pycnocephalus* | Forb | Exotic | Y | Y |
| *Carpobrotus edulis* | Forb | Exotic | Y | Y |
| *Centromadia pungens* | Forb | Native | Y | Y |
| *Cirsium vulgare* | Forb | Exotic | Y | Y |
| *Convolvulus arvensis* | Forb | Exotic | Y | Y |
| *Cotula australis* | Forb | Exotic | Y | Y |
| *Cotula coronopifolia* | Forb | Exotic | Y | Y |
| *Crypsis schoenoides* | Forb | Exotic | Y | Y |
| *Distichlis spicata* | Grass | Native | Y | Y |
| *Dittrichia graveolens* | Forb | Exotic | Y | Y |
| ***Elymus triticoides*** | **Grass** | **Native** | **N** | **N** |
| *Epilobium brachycarpum* | Forb | Native | Y | Y |
| *Eriogonum fasciculatum* | Forb | Native | Y | Y |
| *Eriophyllum confertiflorum* | Forb | Native | N | N |
| *Erodium botrys* | Forb | Exotic | Y | Y |
| *Erodium cicutarium* | Forb | Exotic | Y | Y |
| *Eschscholzia californica* | Forb | Native | Y | Y |
| *Euthamia occidentalis* | Forb | Native | Y | Y |
| *Festuca bromoides* | Grass | Native | Y | Y |
| ***Festuca microstachys*** | **Grass** | **Native** | **Y** | **Y** |
| *Festuca perennis* | Grass | Exotic | Y | Y |
| *Foeniculum vulgare* | Forb | Exotic | Y | Y |
| *Frankenia salina* | Forb | Native | Y | Y |
| *Geranium dissectum* | Forb | Exotic | Y | Y |
| *Glebionis coronaria* | Forb | Exotic | Y | Y |
| *Grindelia stricta* | Forb | Native | Y | Y |
| *Heliotropium curassavicum* | Forb | Native | Y | Y |
| *Helminthotheca echioides* | Forb | Exotic | Y | Y |
| *Heterotheca grandiflora* | Forb | Native | Y | Y |
| *Hirschfeldia incana* | Forb | Exotic | Y | Y |
| *Hordeum depressum* | Grass | Native | Y | Y |
| *Hypochaeris glabra* | Forb | Exotic | Y | Y |
| *Kickxia elatine* | Forb | Exotic | Y | Y |
| *Lasthenia californica* | Forb | Native | Y | Y |
| *Lasthenia glabrata* | Forb | Native | Y | Y |
| *Lepidium didymum* | Forb | Exotic | Y | Y |
| *Lepidium latifolium* | Forb | Exotic | Y | Y |
| *Limonium californicum* | Forb | Native | Y | Y |
| *Lotus corniculatus* | Forb | Exotic | Y | Y |
| *Lupinus succulentus* | Forb | Native | Y | Y |
| *Malva parviflora* | Forb | Exotic | Y | Y |
| *Malvella leprosa* | Forb | Native | Y | Y |
| *Melilotus officinalis* | Forb | Exotic | Y | Y |
| *Mesembryanthemum nodiflorum* | Forb | Exotic | Y | Y |
| *Parapholis incurva* | Grass | Exotic | Y | Y |
| *Phacelia californica* | Forb | Native | Y | Y |
| *Plantago major* | Forb | Exotic | Y | Y |
| *Polygonum aviculare* | Forb | Exotic | Y | Y |
| *Rosa californica* | Forb | Native | N | Y |
| *Salicornia depressa* | Forb | Native | Y | Y |
| ***Salicornia pacifica*** | **Forb** | **Native** | **Y** | **Y** |
| *Salsola soda* | Forb | Exotic | Y | Y |
| *Salsola tragus* | Forb | Exotic | Y | Y |
| *Senecio vulgaris* | Forb | Exotic | Y | Y |
| *Silybum marianum* | Forb | Exotic | Y | Y |
| *Sonchus oleraceus* | Forb | Exotic | Y | Y |
| *Spergularia marina* | Forb | Native | Y | Y |
| *Stipa miliacea* | Grass | Exotic | N | N |
| *Stipa pulchra* | Grass | Native | Y | Y |
| *Suaeda nigra* | Forb | Native | Y | Y |
| *Symphyotrichum chilense* | Forb | Native | Y | Y |
| *Tetragonia tetragonioides* | Forb | Exotic | Y | Y |
|  |  | **Total** | **81 (92%)** | **85 (97%)** |

^1^ At least one ITS sequence in Genbank.

^2^ At least one trnL sequence in Genbank.
