# Supplementary material for: Bird nests as botanical time capsules: DNA barcoding identifies the contents of contemporary and historical nests
Source: PLoS One. 2021 Oct 6;16(10):e0257624. doi: 10.1371/journal.pone.0257624 (PMC8494352; doi:10.1371/journal.pone.0257624)
Supplement: S1 Protocol — (DOCX) [file pone.0257624.s010.docx]

**Bird nests as botanical time capsules: DNA barcoding identifies the contents of contemporary and historical nests.** Rinkert et al.

**S4 Protocol. Creating an ITS reference sequence of *Rosa californica*.**

In order to supplement existing GenBank ITS sequence resources for *Rosa*, we targeted a transitional habitat candidate species, *Rosa californica* (Cooper 1926). Fresh leaves were collected from Terrace Point, Santa Cruz Co., CA, USA (#AMR2021001 deposited at Carl W. Sharsmith Herbarium, San José State University, San José, CA) and stored on ice until DNA could be extracted. DNA extraction from approximately 100 mg of leaflet from #AMR2021001 followed the same procedure as described herein for bird nest materials. To amplify the ITS region, we used ITS5* and ITS26S-25R, reagent amounts, and thermal cycling conditions as described in Whittall (1999). After shrimp-alkaline phosphatase PCR clean-up, the sample was sequenced in forward and reverse directions twice using Sanger sequencing (Sequetech, Mountain View, CA). Individual reads were aligned, primers were removed and the consensus was extracted in Geneious Prime (Kearse et al. 2012). This sequence (GenBank Accession MW686548) was added to the *Rosa* alignment containing sample 29-4 and non-redundant extraction from the top 100 BLAST hits.
